# Ultrasound Pitfalls in a Complex Fetal Cardiac Malformation—Case Report of a New Arteriovenous Central Communication

**DOI:** 10.3390/diagnostics11122398

**Published:** 2021-12-20

**Authors:** Roxana Elena Bohîlțea, Adrian Dumitru, Radu Vlădăreanu, Liana Pleș, Tiberiu Augustin Georgescu, Ioan-Andrei Petrescu, Octavian Munteanu

**Affiliations:** 1 Department of Obstetrics and Gynecology, Carol Davila University of Medicine and Pharmacy, 020021 Bucharest, Romania; vladareanu@gmail.com; 2 Department of Obstetrics and Gynecology, Filantropia Clinical Hospital, 011132 Bucharest, Romania; 3 Department of Pathology, Carol Davila University of Medicine and Pharmacy, 020021 Bucharest, Romania; dr.adriandumitru@yahoo.com (A.D.); tiberiuaugustin.georgescu@gmail.com (T.A.G.); 4 Department of Obstetrics and Gynecology, Elias University Emergency Hospital, 011461 Bucharest, Romania; 5 Department of Obstetrics and Gynecology, Sf Ioan Hospital-Bucur Maternity, 040294 Bucharest, Romania; 6 Faculty of Medicine, Carol Davila University of Medicine and Pharmacy, 020021 Bucharest, Romania; 7 Department of Anatomy, Carol Davila University of Medicine and Pharmacy, 020021 Bucharest, Romania; octav_munteanu@yahoo.com

**Keywords:** fetal arteriovenous malformation, aortic coarctation, ventricular septal defect, 3D ultrasound, Doppler ultrasound, linguo-facial vein, brachiocephalic vein

## Abstract

Cardiac and cardiovascular malformations are of real interest in terms of definition, epidemiology, and means of early diagnosis by imaging. Although ultrasound examination reaches exceptional performance nowadays, unusual pathologies are still exposed to the risk of either incorrect acquired image or misinterpretation by the specialist in a routine scan. Herein, we present a case of a 20-week-old fetus (from an apparently low-risk pregnancy) with complex cardiac and vascular abnormalities, including an arteriovenous malformation along with ventricular septal defect, ductal coarctation of the aorta, aneurysm of a brachiocephalic vein, and dilation of the entire neck and upper mediastinum venous system, and the limitations that were encountered in the process of diagnosis and management of the case.

## 1. Introduction

Understanding the embryology of the fetal venous system is of real interest in terms of ultrasound analyzing the venous anatomy and antenatal diagnosing of cardiovascular anomalies [1]. In the present circumstances, where obstetrics focuses on the more non-invasive and precise methods of prenatal diagnosis, the accuracy of anomalies identification arises from the breakthrough that Doppler ultrasound has made in recent years [2,3,4,5].

Precedent studies describing the development of the fetal venous system focus on the greater vessels in the organism and the ones related to the central circulation (i.e., cava veins) [2,6], and just a few studies address certain segments, such as cervical veins [7]. Hence, abnormalities described in the literature and most encountered in practice mostly refer to fetal circulation malformations of the veins that can conveniently be approached/revealed by ultrasound [6,8,9,10,11,12]. Even though, except for persistent left superior vena cava fetal venous malformations, venous malformations are rare [1]. Most of the cases in the literature refer to cava and azygos venous systems coronary sinuses, brachiocephalic veins, umbilical and vitelline veins, and their derivatives and anomalies, and even lesser are the cases involving pulmonary circulation [1,6,9,13,14,15]. Regarding pulmonary circulation anomalies, veins encounter malposition and atrial connection anomalies [9], while arteries are described to run into defects more often, such as caliber and origin malformations, some of which are included in the description of congenital syndromes [16,17].

Interestingly, arteriovenous malformations (AVMs) are a rather bizarre spectrum of pathology, and most relevant cases mainly involve umbilical arteriovenous fistula [18,19]. Emphasizing the unique cases of AVM field existing in the prenatal and neonatal pathology allows obstetricians to recognize the vital risk regardless of their single occurrence or association with other conditions that could affect the pregnancy and fetal development.

We report a case and a morphological study of a male fetus with an AVM between the left pulmonary artery and an aneurism of the left brachiocephalic vein with multiple simultaneous vascular anomalies and cardiac development defects.

## 2. Case Presentation Section

We report the case of a 31-year-old Caucasian woman, gravida 3, para 1, who was referred after a second trimester fetal anatomy screening at 20 weeks gestational for a suspicion of a complex fetal cardiac malformation, for which several specialized opinions tried to reach consensus.

The obstetrical history of the patient includes a previous Caesarian section with a normal course of parturition and a spontaneous miscarriage. The current pregnancy presented a low risk for aneuploidy according to the performed cell-free fetal DNA test. The classical karyotype performed after the abortion did not reveal any chromosomal abnormalities.

### 2.1. Ultrasound Findings

Previous ultrasound evaluations were incongruent and reported the following findings:

- an isolated aortic arch anomaly (supposedly aneurysmal dilation from which the left common carotid artery emerges) and coarctation of the aorta with the anterograde flow;
- ventricular septal defect, coarctation of the aorta, and a vascular formation located superior from the aortic arch with the appearance of an arteriovenous fistula;
- aneurysmal dilation located above the pulmonary trunk bifurcation and a dilated left common carotid artery with a retrograde flow;
- minor ventricular septal defect with a normal ductus venosus triphasic flow.

**Figure 1:**
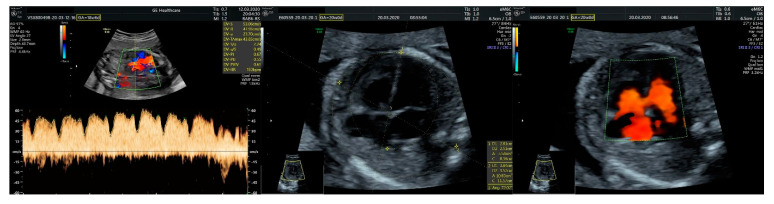
(**Left**): normal triphasic flow of the ductus venosus; (**middle**): mild cardiomegaly and a common four-chamber aspect of the heart; (**right**): membranous ventricular septal defect.

**Figure 2:**
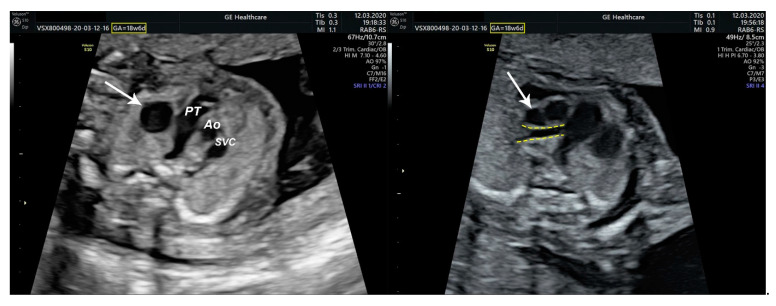
Transversal sections through the fetal mediastinum; Three-vessel view. White arrow—aneurysmal structure located above the emergence of the pulmonary artery; Yellow dashed lines—communication between the aneurysmal structure and either the pulmonary trunk or the left pulmonary artery; PT—pulmonary trunk; Ao—(ascending) Aorta; SVC—superior vena cava.

**Figure 3:**
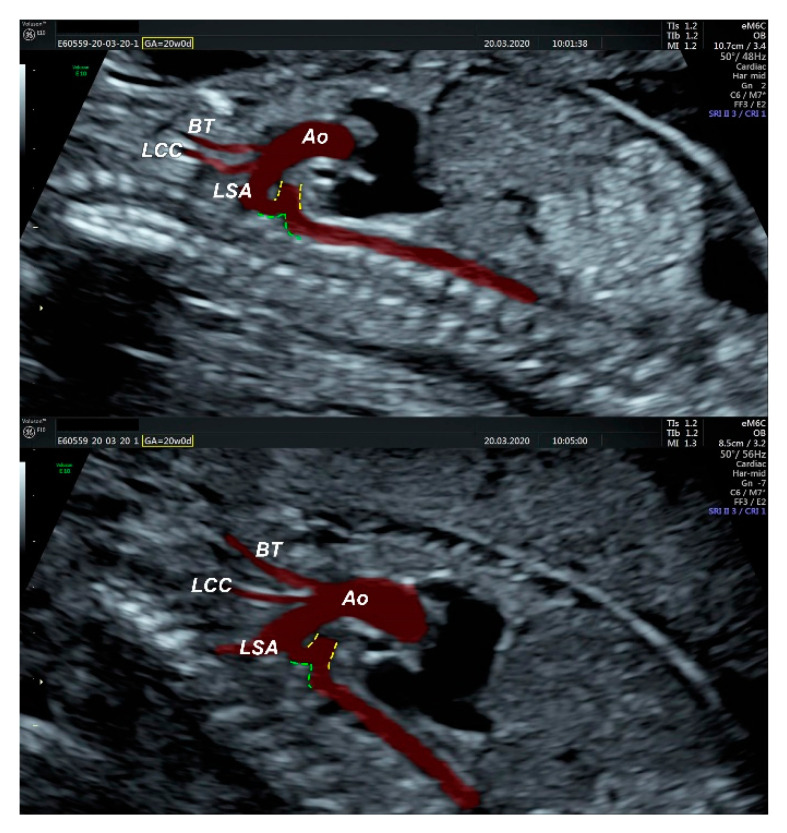
Sagittal section through the fetal mediastinum. Ao—aorta; BT—brachiocephalic trunk; LCC—left common carotid artery; LSA—left subclavian artery; Yellow dashed lines highlight the patent ductus arteriosus; Green dashed line—ductal coarctation of the aorta.

**Figure 4:**
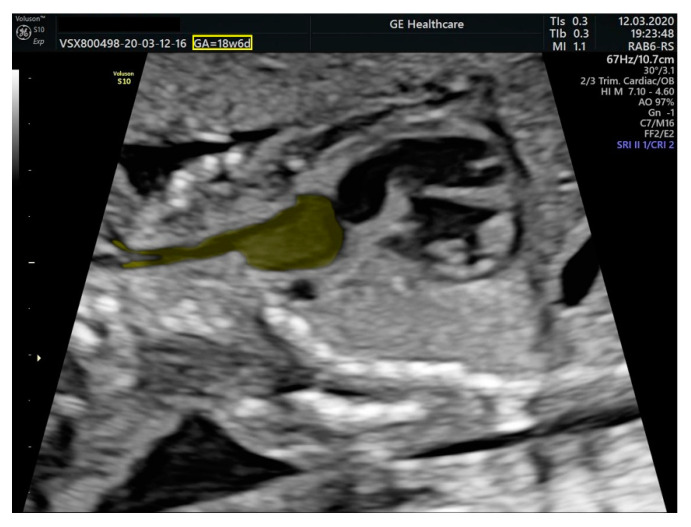
Sagittal section through the fetal cervical region and mediastinum. The aneurysm is located above the base of the heart and gives rise to a vascular structure that dichotomously branches in the cranial half of the cervical region.

**Figure 5:**
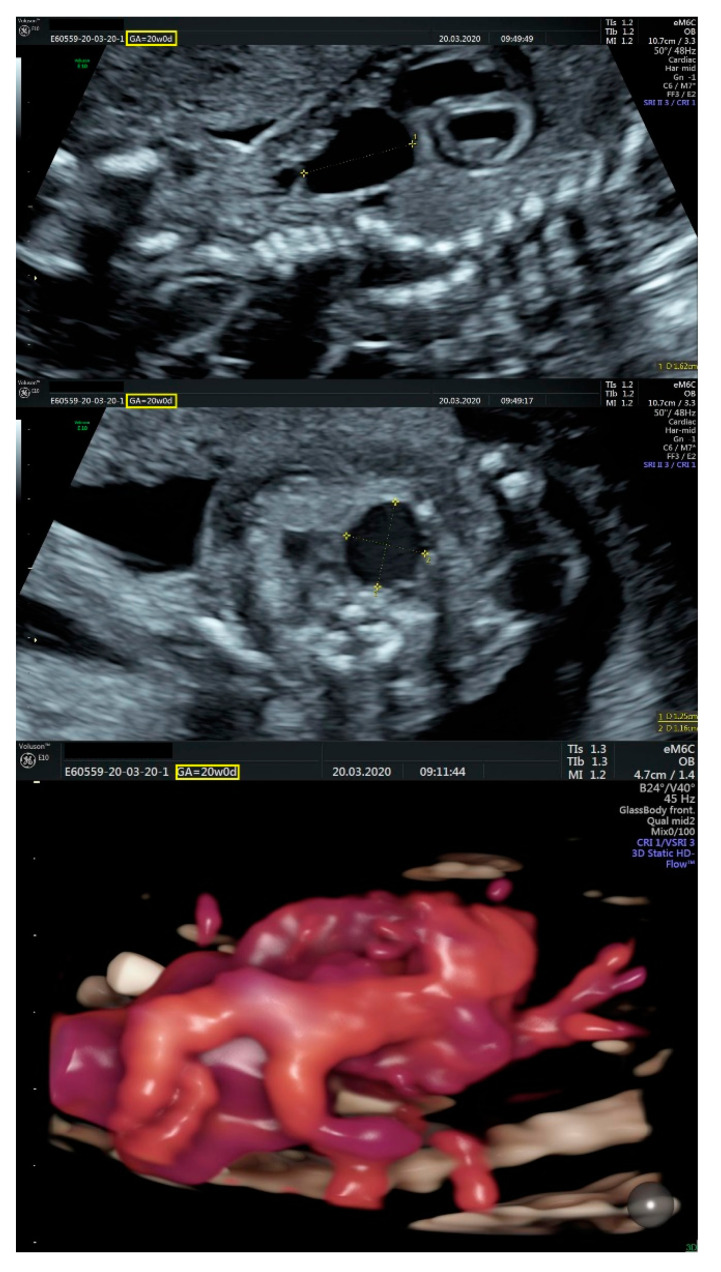
(**Top** and **middle**): ultrasonographic measurements of the aneurysm in sagittal (**top**) and transversal (**middle**) incidences; (**bottom**): 3D reconstruction of the mediastinal vessels.

**Figure 6:**
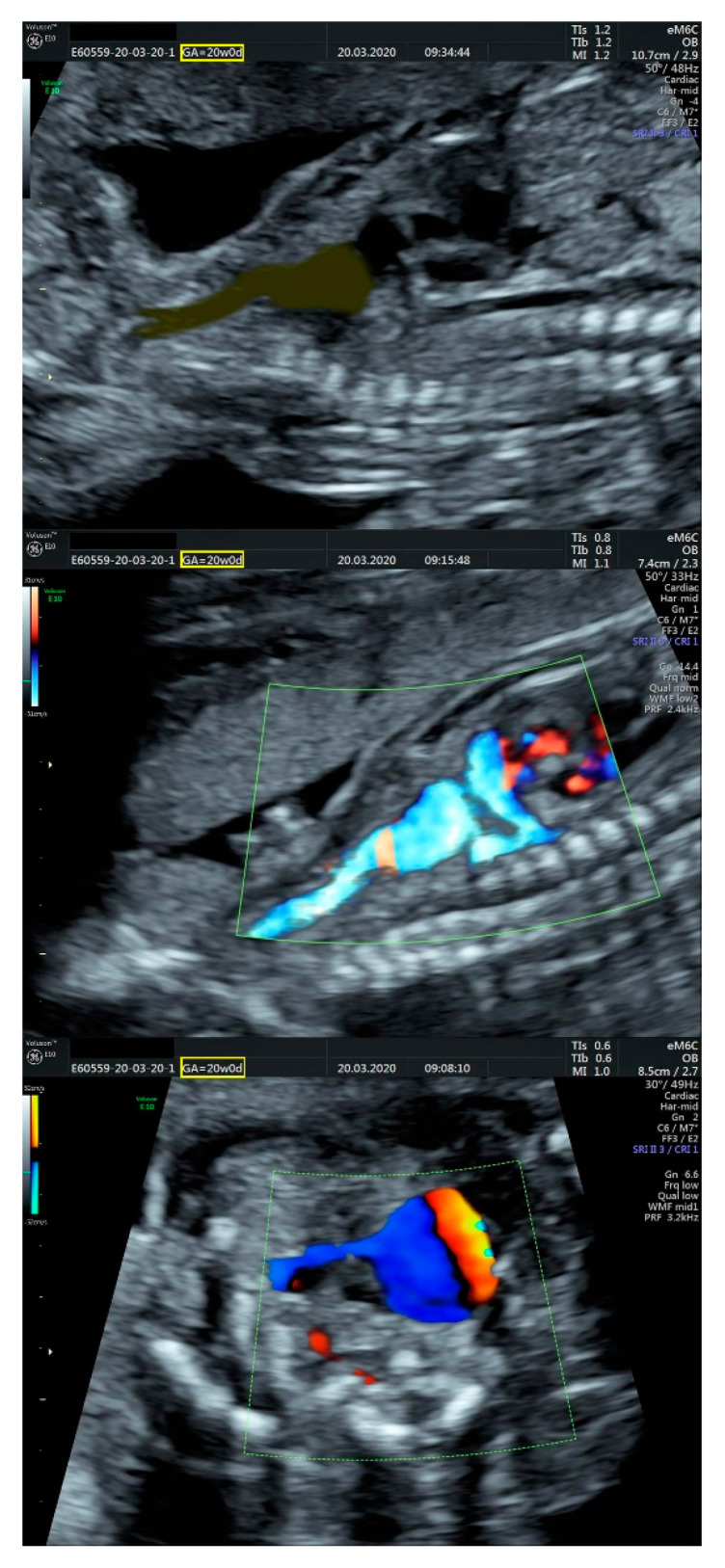
(**Top** and **middle**): sagittal section and Doppler examination of the aneurysm and the vascular pattern which bifurcates towards the cervical region; (**bottom**): turbulent Doppler flow highlighting the communication between the aneurysm and pulmonary trunk/left pulmonary artery.

We performed fetal echocardiography, which demonstrated a mild cardiomegaly with a left deviated 72-degree heart axis, normal aspect of the four-chamber view, a small membranous ventricular septal defect, and ductal aortic coarctation; the ductus venosus flow was normal (Figure 1, Figure 2 and Figure 3). In addition, we identified an aneurysmal structure measuring 1.63/1.25/1.16 cm with turbulent Doppler flow, situated above the emergence of the pulmonary trunk and continued by a dilated vascular structure that bifurcates in the cervical region; the aneurysm seemed connected to the left pulmonary artery as well. A dilated left subclavian artery was also suspected (Figure 4, Figure 5 and Figure 6).

In the context of complex cardio-vascular malformations, the patient requested the termination of the pregnancy by drug-induced abortion.

### 2.2. Dissection

The hands-on dissection of the fetus revealed a set of abnormalities that could stand as an anatomical basis for what has been found during the ultrasound examination.

**Figure 7:**
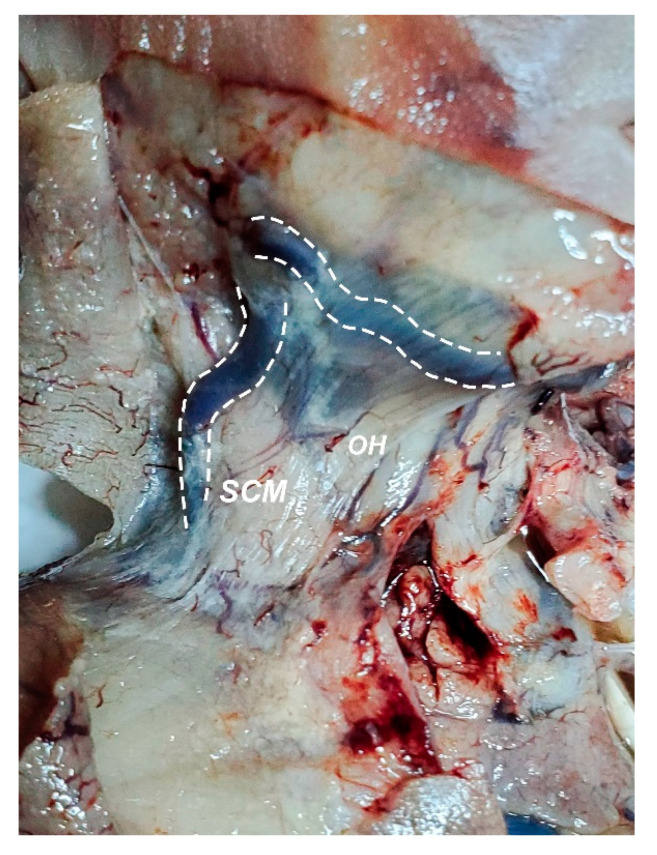
Neck, right view; skin was removed. Dashed lines highlight linguo-facial trunk (up) and external jugular vein (left), which are visible through the transparency of the platysma; Bluish coloration of the carotid triangle is due to the dilation of the internal jugular vein; SCM—sternocleidomastoid muscle; OH—omohyoid muscle.

**Figure 8:**
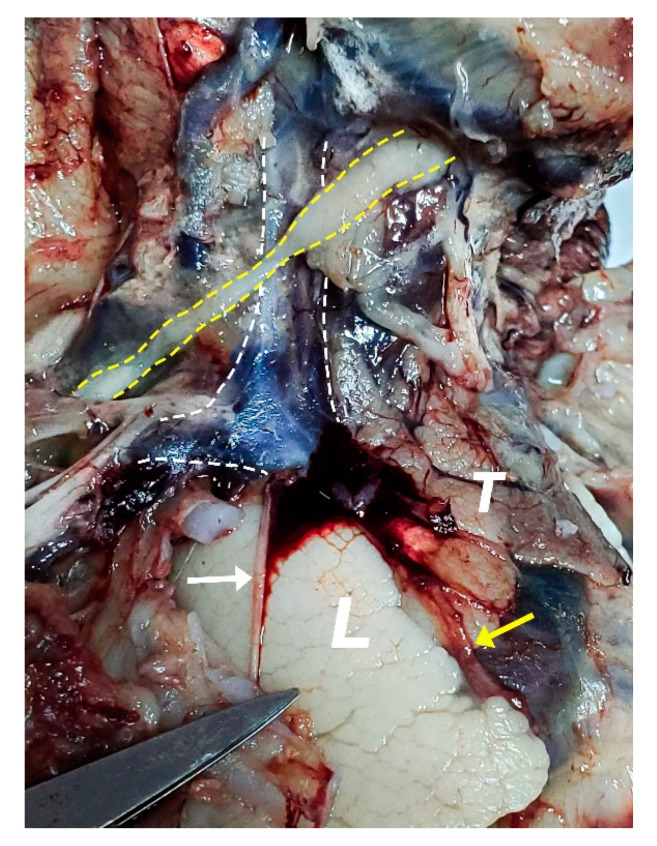
Neck, thoracic cavity, right-anterior view; platysma and SCM were removed. White dashed lines highlight the right internal jugular vein, right subclavian vein, and right brachiocephalic vein. Dilation of the right internal jugular vein and the right subclavian vein. The conflation of the two into the right brachiocephalic vein is wider in caliber as well. Yellow dashed lines highlight the omohyoid muscle, running over the internal jugular vein. L—right lung; T—thymus; White arrow—vagus nerve; Yellow arrow—phrenic nerve.

**Figure 9:**
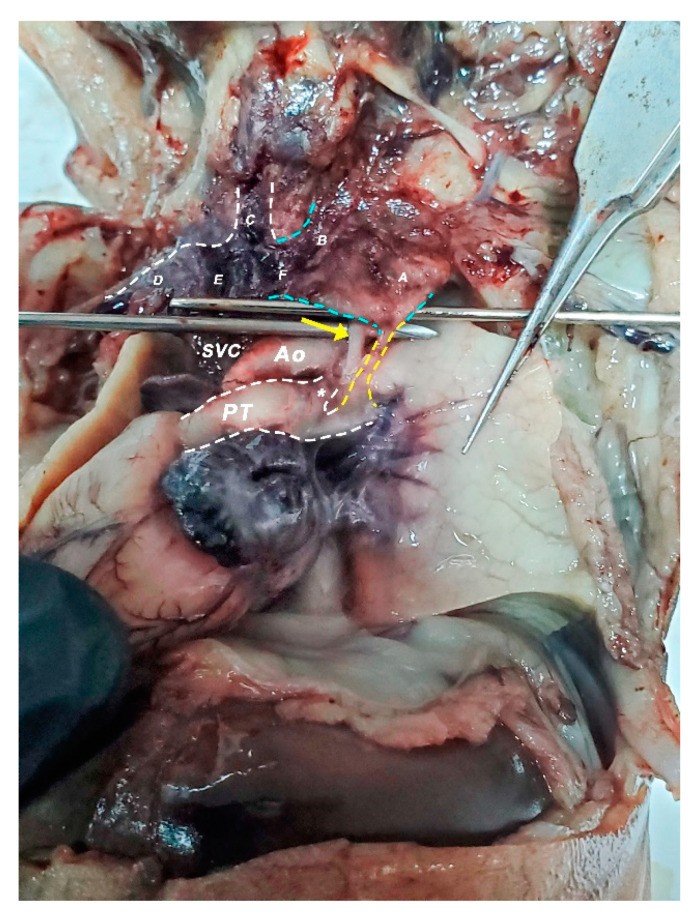
Heart, mediastinal vessels, oblic-left view; heart was pulled towards the right in order to draw attention to the arteriovenous anastomosis. Yellow dashed lines highlight the anastomosis between the left pulmonary artery and left brachiocephalic trunk. Blue dashed lines outline the contour of the enlarged left brachiocephalic trunk. A—left subclavian vein; B—left internal jugular vein; C—right internal jugular vein; D—right subclavian artery; E—right brachiocephalic trunk; F—left brachiocephalic trunk. SVC—superior vena cava; Ao—aorta/aortic arch; PT—Pulmonary trunk; Yellow arrow—vagus nerve.

The first and the most pronounced aspect was the distention of the whole venous system of the neck and mediastinum. The specimen presented a linguo-facial vein that described a rather sinuous pathway alongside the inferior margin of the mandible (Figure 7). Both the linguo-facial vein and the external jugular vein appeared with a markedly increased caliber, around 4–5 times larger than expected for this gestational age. Both left and right jugular veins and the right subclavian vein were assessed as three times larger than usual, respecting the normal relations to the neighboring structures (Figure 8 and Figure 9).

The confluence between the left jugular and subclavian vein into the left brachiocephalic vein was observed to be very dilated to superior and inferior, extending above the superior margin of the omohyoid muscle as well as below the inferior concavity of the aortic arch. Moreover, on the inferior side of the enlarged brachiocephalic vein, a vessel could be observed descending lateral to the left vagus nerve and communicating with the left pulmonary artery. The left pulmonary artery was observed to be dilated as well, around twice as normal (Figure 9).

**Figure 10:**
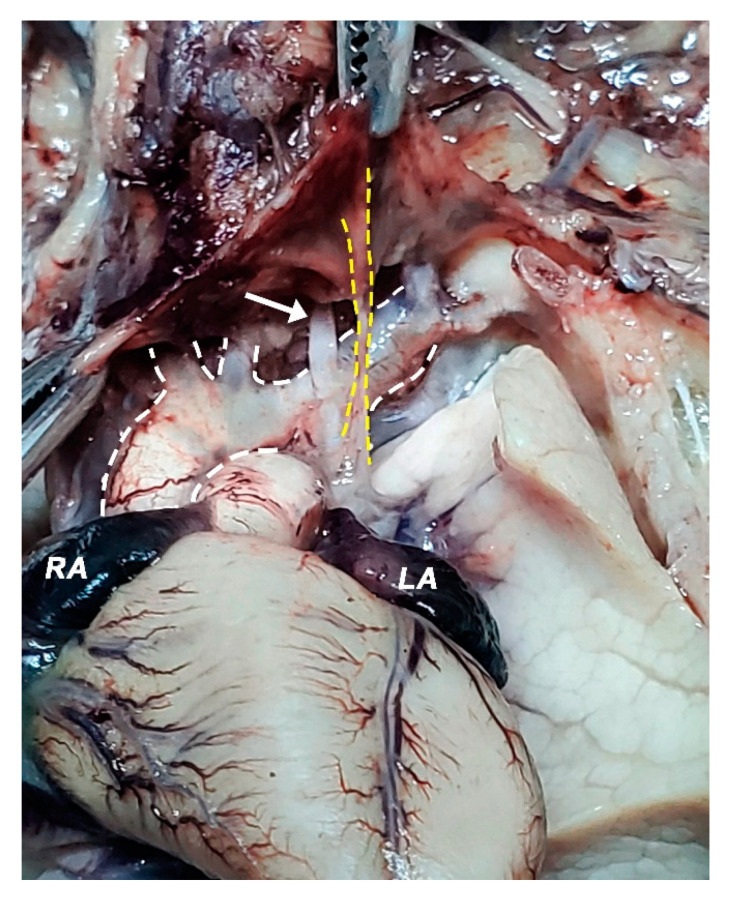
Heart, the greater vessels of the heart, front view; brachiocephalic veins set aside (forceps). White dashed lines highlight the aortic arch and the vessels emerging from it; the left subclavian artery appears dilated, its diameter being comparable with the caliber of the aortic arch itself; Yellow dashed lines highlight the arteriovenous anastomosis (top forceps) descending anterior to the left subclavian artery and left from the vagus nerve (white arrow); LA—left auriculum; RA—right auriculum.

**Figure 11:**
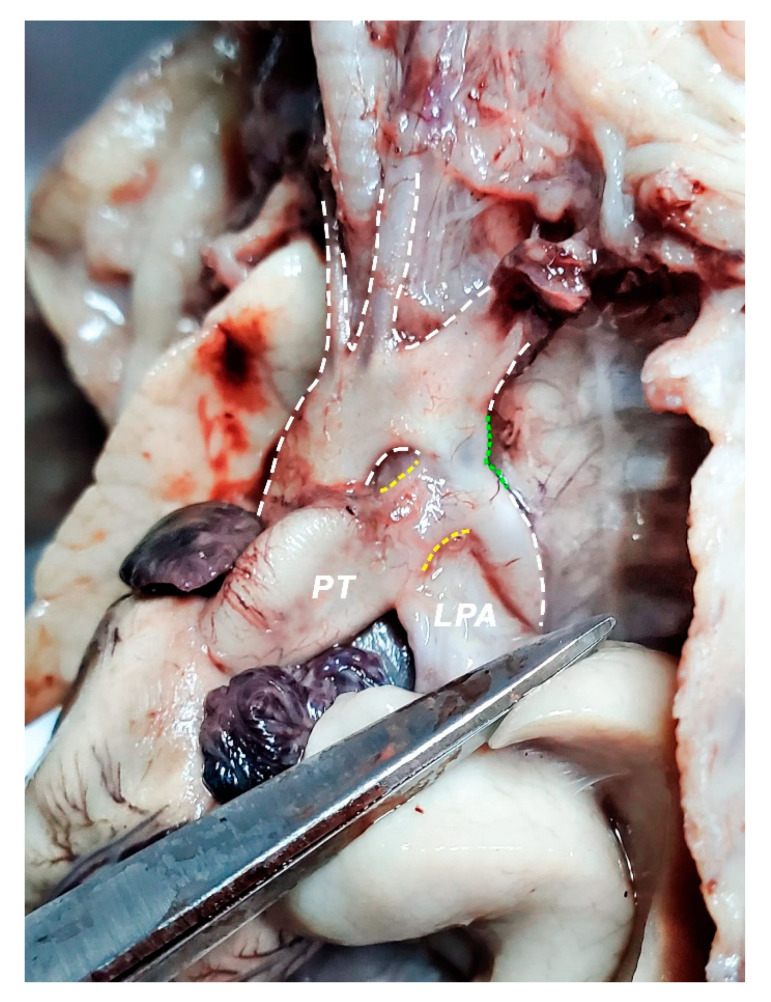
Mediastinum, heart and greater vessels; left view; left lung was pulled inferiorly in order to highlight left pulmonary artery (LPA) and ductus arteriosus (yellow dashed lines). Green dashed line highlights the aortic coarctation, situated distally from the left subclavian artery emergence situs. Ductus arteriosus appears wide in dimension; it emerges on the anterior aspect of the pulmonary trunk (PT) and ends at the level of the aortic narrowing. LPA emerges posterior from the ductus arteriosus and is put into tension by the dissection instrument in the picture. Thus, a right angle between PT and LPA is noticeable in the figure due to the traction we exerted on the left pulmonary hilum. The left subclavian artery gives the impression of a rather terminal branch of the aorta, appearing almost as wide as the aortic arch itself.

Regarding the great vessels of the heart, there are some anomalies to be discussed. A narrowing of the aortic arch was identified distally to the emergence of the left subclavian artery (Figure 9 and Figure 10). A large, patent ductus arteriosus was found, ending right at the narrowing level observed in the aortic arch (ending right at the coarctation level) (Figure 11). The left subclavian artery was dilated as well, sizing as much as the ascending aorta and the aortic arch, creating the illusion of a terminal branch rather than a lateral one (Figure 5 and Figure 6).

Heart analysis concluded no distinct changes in heart architectural formation for this gestational age. Atrioventricular and ventriculoarterial concordance was noted. Atria and ventricles were increased in relation to the mediastinum. Surprisingly for an aortic coarctation, the right atrium was not found to be enlarged.

## 3. Discussion

The particularity of the reported case is represented by a complex cardio-vascular malformation including: cardiomegaly, membranous ventricular septal defect, ductal coarctation of the aorta, brachiocephalic vein aneurysm with a patent hemodynamic arteriovenous anastomosis between the brachiocephalic trunk and the left pulmonary artery, and dilation of the neck venous system. Hence, if we are to consider any of these diagnosis criteria, any attempt to correlate the case to a particular syndrome would be rather futile.

The prenatal correct diagnosis is difficult in such a complex condition, and the different opinions confirm that issue. The assumption of a left common carotid artery emerging from the ultrasonographically objectified dilation, as well as the Doppler retrograde flow, was possible due to its trajectories and relations and its bifurcation in the cervical region. Indeed, the common carotid artery has an ascending course and bifurcates into the internal and external carotid arteries at the level of the Carotid Triangle, but the common facial vein—also known as linguo-facial vein or thyro-linguo-facial vein—respects the same pathway and relations [20,21,22]. However, we managed to capture the three vessels emerging from the aortic arch in the same plane (Figure 6), concluding that the only enlarged artery in the mediastinum was the left subclavian artery, as described in Figure 6, Figure 10 and Figure 11.

Prenatal evaluation of the fetal venous system can be performed only by ultrasound scans, including B mode, Doppler color, and power Doppler. The guidelines and protocols address the main fetal veins (cava vein system, pulmonary veins, and intra-abdominal veins, including umbilical, portal vein, and ductus venosus) considering their impact on cardiac hemodynamic and fetal outcome. Their congenital anomalies are the most frequent and relatively standardized ways to assess those veins during ultrasound training process. All of the above-mentioned factors limit the evaluation capability of the average ultrasonographer to correctly diagnose such anomalies. As a consequence, better training for carefully evaluating the entire fetal venous system, including head and neck veins, could overcome those limitations.

During the autopsy assessment, the dilation of the internal jugular vein and the confluence between this vein and an enlarged linguo-facial vein were identified. This finding confirms that any retrograde flow described through the left common carotid artery was obtained confusedly. On this occasion, it is appropriate to note that anatomy plays a tremendous role in Imagistics as a field, and with this knowledge, differential diagnosis can be guided in a rather holistic manner. However, when addressing the learning curve in fetal ultrasound diagnosis, the focus is put somewhat towards the ability to recognize pathologic features rather than to acquire the skill of extensively discerning by means of orientation and echostructure [23,24,25]—ergo the criticism.

We consider that the enlarged brachiocephalic trunk appeared as an aneurysmal dilation in the ultrasound, taking into account that an arteriovenous communication in both ultrasound assay and post-abortion dissection was encountered (Figure 2, Figure 5 and Figure 10). The ductal aortic coarctation and the large, patent ductus arteriosus also had the same proportions in our ultrasound and anatomic appraisal (Figure 6 and Figure 11). The quantification of the cardiomegaly was problematic during the autopsy since the lungs were collapsed and the thoracic cavity was developed accordingly.

The case also asserts its distinctiveness by the way in which the venous malformation is presented. The arteriovenous anastomosis and the whole supracardiac venous system enlargement are rather isolated events, bearing in mind that literature is pretty scarce in the field of venous malformations [14,26] and that most of the reported cases were abdominal veins abnormalities [27].

Although the diagnosis is initially suspected by the abnormal image of the standard sections (i.e., their vessels and trachea view) and confirmed by color Doppler and power Doppler, more modern techniques can be useful in order to depict the vessels tracts and the flow inside better. The useful tools could be Doppler Radiant flow, HD-live flow, and HD-live flow silhouette, which are appropriate for assessing the fetal cardiac anatomy and vessels and prenatal diagnosis of a congenital anomaly.

A normal triphasic flow of the ductus venosus was somehow unexpected to see in the context of such vascular abnormalities, which could come into disagreement with literature and ultrasound guidelines [28,29,30]. This could only be conceivable by a better understanding of the fetal circulation and hemodynamics, raising the supposition that in this case, the normal ductus venosus flow was due to the compensatory effect of neck venous system dilation, giving the enlarged left brachiocephalic vein the role of a capacitance vessel and the unaffected compliance of the right heart. However, hemodynamics could also explain whether it is the increased pressure of the left pulmonary artery or the venous circulation towards the fetal heart that caused the brachiocephalic trunk aneurysm, as well as why there was no right jugular vein dilation.

Hemodynamic studies of the abnormal vessels can help with the differential diagnosis, in our case, to differentiate a dilatation as a part of the Common Carotid artery (as was initially considered) or as a part of a vein. The specific three-wave pattern of the vein, which also has a low velocity, is clearly different from the biphasic pattern of an artery. Moreover, the consequences of the arteries-venous fistula depict a striking turbulent circulation with a high grade of aliasing, which is also an indirect sign for such malformation. Since the anomaly already exists, the primary prevention cannot be applied, but close monitoring of the fetal cardiac function, including assessment of the cardiac hemodynamic, is useful in order to prevent fetal deterioration in cases when the pregnancy is ongoing. We plan to implement future studies regarding hemodynamic parameters that could aid the differential diagnosis between the Common Carotid artery with its branches and internal jugular vein with its tributaries, being aware of until this report no other arteries-venous fistula conditions have been described at this level, so we have a new start-point study with the advantage of anatomist point of view.

## 4. Conclusions

The early antenatal diagnosis of a congenital complex cardiovascular malformation can only be made by thoroughly understanding the embryology of the fetal circulation and the overall anatomy of the human body. The case we presented is unusual because of the complex abnormalities, which are difficult to lab into any routine protocols used in obstetric practice, and draws attention to the significance of ultrasound expertise and anatomy apprehension in order to improve correct diagnosis and proper counseling in uncommon complex conditions.

## Data Availability

The datasets used and analyzed during the current study are available from the corresponding authors on reasonable request.
